# Epidermal Growth Factor Methods and Protocols

**DOI:** 10.1038/sj.bjc.6603658

**Published:** 2007-03-20

**Authors:** S K Chan, W J Gullick

**Affiliations:** 1 University of Kent, Canterbury, UK

Since the discovery of the epidermal growth factor more than 40 years ago by Stanley Cohen and colleagues, scientific research in this area has expanded to near exponential rates. The epidermal growth factor receptor family and its associated ligands have revealed much in terms of the molecular biology of receptor tyrosine kinases and definitely proved the first link of an activated oncogene to malignant disease. On the basis of this bedrock of science, the pharmacological development of targeted therapies against this signalling system has proved to be one of the most important areas of translational research over the last two decades. Now in the clinic, agents directed against the epidermal growth factor receptor are hugely important tools in the treatment of several different solid tumours. Ongoing clinical trials seek to refine the use of these therapies and further scientific research reveals additional applications such as the use of soluble receptor.

An impressive set of experts active in the field have contributed towards the 14 chapters in this book, across a spectrum from epidermal growth factor receptor protein isolation to complex techniques such as sensitive assay for monoubiquitination of receptor tyrosine kinases. A comprehensive, yet succinct introductory chapter provides a highly readable historical perspective to those coming in this field of study. An entertaining final chapter underlines clinical and pharmacological issues in the use of monoclonal antibodies and small molecule tyrosine kinase inhibitors against this signalling system. Each chapter is well organised with a summary section followed by a brief introduction, outlining how the technique is useful while explaining its relevant application for epidermal growth factor. Materials and equipment are logically and meticulously listed and the methodology is provided with sample results using clear diagrams and simple figures. An impressive notes section at the end of each chapter, drawing upon the authors’ expertise, is sufficiently detailed lending highly useful practical advice at the cutting edge, as if the expert was present in the very same laboratory.

The editors are to be applauded for the structure, presentation and choice of methods presented which are relevant to the ongoing research. The references are comprehensive and clearly laid out. The information is easily digestible and very hands-on and would provide a useful laboratory resource for planning and reference. For the clinicians, this book would provide an interesting tour into much of the biology of epidermal growth factor receptor and its increasing relevance to clinical application. Overall, this is a well-planned and well-written book covering a variety of techniques applicable to the study of epidermal growth factor receptor and would find a place in those laboratories both already established and those starting out in this ever expanding field of research.

